# Learning from the Kenyan experiment: key takeaways for implementing managed equipment services in developing countries

**DOI:** 10.3389/frhs.2025.1361261

**Published:** 2025-04-28

**Authors:** Ephantus Njagi, Keneth Iloka, Sasha Wawira, Laban Thiga, Nicholas Muraguri

**Affiliations:** ^1^ Department of Human Pathology, University of Nairobi, Nairobi, Kenya; ^2^ School of Engineering and Technology, Kenyatta University, Nairobi, Kenya; ^3^ Adam Smith Business School, University of Glasgow, Glasgow, United Kingdom; ^4^ Ministry of Health, Nairobi, Kenya

**Keywords:** managed equipment services (MES), public-private partnership (PPP), public procurement, universal health coverage (UHC), healthcare system

## Abstract

**Background:**

In 2015, the Kenyan government signed 7-year contracts with 5 Original Equipment Manufacturers (OEMs) to improve healthcare accessibility and equity. The OEMs were to supply, install, maintain, and replace equipment and provide user training for 98 hospitals across Kenya's 47 counties through a Managed Equipment Services (MES) arrangement. This paper highlights the planning, procurement, and implementation of Kenya's first comprehensive MES arrangement.

**Methods:**

Retrospective review of the implementation process drawing data from program databases, reports, and other relevant sources.

**Results:**

The MES program was successfully implemented in Kenya for the first time to upscale specialised health infrastructure and expand critical healthcare services across the 47 counties. Previously unavailable services in the county's hospitals, such as dialysis, were set up in 49 hospitals, critical care units in 11 hospitals, and theatre, sterilisation, and imaging services were expanded in 98 hospitals. The program provided reliable equipment installation and maintenance, increased healthcare workers' capacity through training, and created a more conducive working environment. Key lessons learned include importance of defining detailed equipment specifications, ensuring comprehensive stakeholder engagement, and allowing sufficient time for assessment and implementation. Challenges encountered were prolonged procurement process, insufficient stakeholder buy-in, and delays in implementation.

**Conclusions:**

We have described our experience of planning, procurement, and implementation processes and the lessons learned from a large and comprehensive MES project in Kenya. The MES process is intricate and time-consuming, requiring a team of skilled professionals. Prior to beginning the MES design, a well-planned hospital assessment can alleviate potential obstacles. Despite financial limitations, MES arrangement has the potential to enhance significantly healthcare services, particularly in low- and middle-income nations.

## 1 Introduction

Globally, the demand for adequate and equitable healthcare services has increased due to population increase and the high burden of diseases (1, 2). In Kenya, the population has increased from 15.3 million in 1979 to 47.6 million in 2019, an increase of 2.5-fold over 30 years (3). The disease burden has also increased due to the high prevalence of communicable and rising incidence of non-communicable diseases (NCD), with NCD-related deaths increasing from 27% in 2014 to 39% in 2019 (4, 5). The rapid population growth and high burden of diseases have put tremendous pressure on Kenya's healthcare system, increasing demand for accessible and affordable health services beyond the system's capacity. As a result, the Kenya government has developed various strategies to provide adequate and equitable healthcare services of sufficient quality, consistent with the needs of the population, as guided by Kenya's 2010 Constitution and Vision 2030, which seeks to invest in the people to improve their quality of life (6, 7). In addition, the government has developed various policies and guidelines to provide Universal Health Coverage (UHC) that can sustain a healthy population and meet the United Nations' Universal Declaration of Human Rights commitment and sustainable development goals (8–10).

Despite the development of policies, implementing UHC has been a challenge in Kenya due to an insufficient budgetary allocation to provide healthcare to the increasing population. Although the Kenya government's healthcare budget increased from 7.8% in 2012 to 9.1% in 2020, the trend falls below the 2001 Abuja Declaration target of 15% (11–14). Inadequate investment in health has led to severe implications for the country's health system and capacity to meet its vast needs. There exists inequity in geographical access to health facilities, with most of the hospitals being found in urban areas, leaving populations in rural and marginalised areas, where close to 70% of the population reside, to depend mainly on dispensaries and health centres (15–17). Access to well-equipped health facilities varies widely, with referral hospitals primarily found in large urban towns providing the best healthcare (18–20). Within the hospitals, inadequate availability, and disparity in access to vital equipment needed to deliver high-quality healthcare exist (21, 22). A lack of prioritisation of the available medical equipment maintenance results in disuse (23–25). Added to this is a shortage of skilled human resources and uneven distribution of the available healthcare workers, mostly clustered around large towns where referral hospitals are found (26, 27).

Disparities in access to specialised healthcare services, inadequate equipment maintenance, and the lack of fit-for-purpose medical equipment have long hindered equitable healthcare delivery in Kenya. To address these challenges, the Kenyan government implemented the Managed Equipment Services (MES) model in 2014, adopting a public-private partnership (PPP) approach to enhance service availability across 98 hospitals nationwide. MES was designed to provide a sustainable solution by integrating the supply, installation, testing, maintenance, and replacement of medical equipment, alongside capacity-building through training, over a seven-year contractual period. This model aimed to eliminate the need for an immediate large capital outlay while ensuring continuous functionality and service delivery (28).

Kenya's experience with MES offers valuable insights into the feasibility of large-scale healthcare equipment management through PPPs. The nationwide implementation has demonstrated the potential of MES to improve service delivery, optimise resource utilisation, and strengthen healthcare infrastructure. By documenting the planning, procurement, and implementation processes, this study presents lessons from Kenya's MES experience that may inform other countries exploring similar healthcare financing and service delivery models.

## 2 Methods

### 2.1 Setting

Kenya's healthcare delivery is devolved into 47 county governments, with the national government's responsibility being health policy and national referral hospitals (6). The healthcare system is provided through a network of nearly 13,000 health facilities distributed across 47 counties. Public facilities account for 46%, private for-profit 43%, faith-based 8%, and NGO-run 3% (17). The system is structured in a stepwise approach; level 1 is community health units, level 2 is dispensaries and clinics comprising 76% of all the health facilities, and level 3 is health centres and maternity homes comprising 17%. The remaining 7% are levels 4 and 5, sub-county and county referral hospitals, and level 6, national referral hospitals (18). While most public and faith-based facilities serve rural areas, Level 5 and 6 public hospitals, along with private for-profit facilities, are concentrated in densely populated urban centres. As one goes up the hierarchy from level 1 to level 6, the service complexity and the technical capability of human resources and equipment all increase. In this system, patients move from one level to the next, using a referral system with complicated cases referred to a higher level (18).

The health system is financed by revenues collected by national and county governments through taxes and donor funding, the National Hospital Insurance Fund (NHIF), and private health insurance companies through members' contributions and out-of-pocket spending at points of care (29, 30). Health insurance coverage is still insignificant, although an upward trend from 6.7% in 2017 to 8.4% in 2019 has been noted (31–33).

### 2.2 Need analysis

In 2013, many stakeholders began raising concerns about the poor physical infrastructure and equipment in most health facilities and the disparities in access to specialised health services across the country. Members of Parliament expressed their concern through a senate motion, moved and adopted on 26th June 2013, compelling the national government to appropriately equip levels 4 and 5 hospitals in each of the 47 counties. The Health Sector Intergovernmental Consultative Forum, which includes representatives from both levels of government, also met in October 2013 and agreed to bridge the gap in healthcare access and inequity by equipping 98 hospitals with modern equipment. All 47 counties were requested to select two hospitals for strengthening, a county referral and a sub-county hospital, to be added to 4 national referral hospitals. In February and March of 2014, in consultation with the 47 county governments, the Ministry of Health (MoH) conducted a needs assessment in the selected 98 hospitals across the country. The assessment aimed to determine the status of the selected hospitals' infrastructure, equipment, and human resources. The assessment showed a lack of critical equipment, with those available being too old and characterised by frequent breakdowns due to lack of maintenance. In addition, there was an insufficient supply of healthcare workers and limited availability of essential services such as dialysis, intensive care services, and mammography. The findings were in line with the previous assessments, including the Kenya Service Availability and Readiness Assessment Mapping (SARAM) (34). The assessment result was used to develop a list of equipment needed to enable the selected hospitals to provide vital services.

### 2.3 Procurement planning

In June 2014, the MoH appointed a team of medical and biomedical engineers and financial and legal experts from public and private institutions to support the MoH in acquiring equipment for the 98 selected hospitals. First, the team reviewed and grouped the equipment list into 7 lots. The scope of each lot was dedicated to developing and supplying all equipment needed to establish fully functional services for theatre (lot 1), CSSD (lot 2), laboratory categories 1 and 2 (lots 3 and 4), dialysis (lot 5), intensive care (lot 6), and imaging (lot 7). Then, based on the standard of the selected services, the team developed technical specifications of all the equipment needed to set up each service.

The team reviewed the available budgetary allocation, and the estimated capital outlay needed to acquire the identified equipment using different options. The options considered were traditional procurement, leasing, and MES (35–39). MES was chosen due to the funds allocated being insufficient for traditional procurement methods or leasing equipment costs. The team developed a draft MES contract and 26 schedules defining specific MES scope and expected services. The contract covered critical features, including equipment replacement plans, partnership management, service specifications, implementation timeframe, maintenance, invoicing, payment mechanisms, and performance parameters. This was used to advertise the tender for supplying, installing, testing, maintaining, and replacing medical equipment and associated training through MES.

### 2.4 Selecting the contractors

The acquisition process followed the procurement procedures set out by the Kenya Public Procurement and Disposal Act No. 33 of 2005 and the Public Procurement and Disposal Regulations of 2006 (40, 41). The tender was advertised in July and August 2014 for Original Equipment Manufacturers (OEMs) with strong finances and a willingness to establish and maintain a local distributorship. The tender aimed to supply, maintain, and replace equipment and offer software updates as needed. During the bidding process, 219 questions were received from bidders, and responses were sent 30 days before the tender's closure. In September 2014, after a bidding validity period of 60 days, 30 companies responded to the tender, submitting tender documents which were opened in the presence of 45 bidders' representatives.

A team appointed by the Ministry of Health conducted the evaluation of tender documents in accordance with Kenya's public procurement regulations (40, 41).

The evaluation followed a structured process, beginning with a mandatory compliance check to determine whether bidders met the basic eligibility criteria. A significant proportion of submissions did not progress beyond this stage due to non-compliance with the outlined requirements. Only a fraction of the bidders met the mandatory criteria and proceeded to the next phase, where their technical proposals underwent a detailed assessment to evaluate their capacity to deliver the specified equipment and services.

Secondly, a predetermined list of technical elements was used to perform a detailed technical evaluation for the bidders responsive to the mandatory requirements. A scoring system for each element was applied, and those who scored below 80% were disqualified. This predetermined cut-off ensured that any successful bidder could fulfil the Kenya Government's essential requirements. Thirdly, a financial evaluation was performed on bids with a technical score above 80%. The financial assessment verified that all equipment and services included in the tender were priced, along with maintenance for a 7-year contract period, which also covered applicable transport costs and taxes.

**Figure 1:**
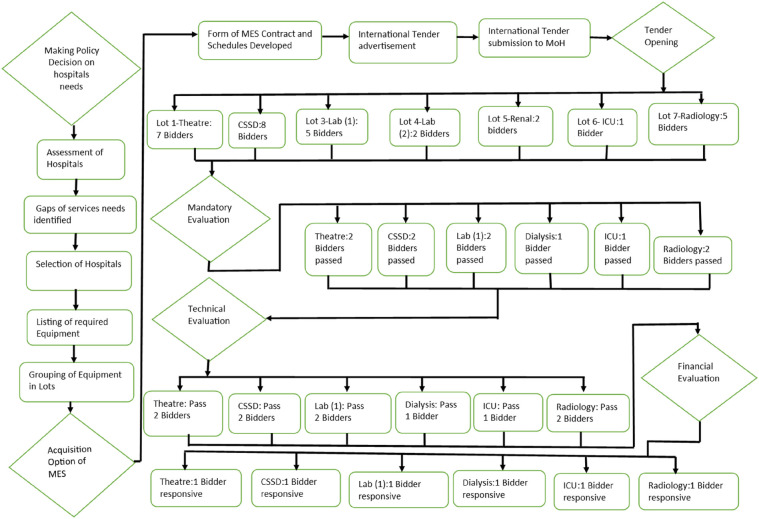
Schematic illustrating managed equipment services acquisition processes.

The tender processing committee took 24 days to evaluate all the bids due to the complexity of the tender and the number of tender documents submitted. Out of the 30 submissions received, 6 bidders (20%) reached the final negotiation stage after meeting the mandatory, technical, and financial criteria. A formal written award notification was sent to the 6 bidders, with each bidder being required to send a notice of acceptance of the offer. One bidder did not accept the notification award, and their bid was not progressed. Figure 1 illustrates the Managed Equipment Services acquisition processes.

Between December 2014 and January 2015, a government team from multiple agencies conducted due diligence on all the bidders who had accepted the written award notification. The team evaluated the bidders' manufacturing, financial, and prior MES performance capabilities. A value-for-money analysis was conducted to assess the market reasonableness of the tender prices and variances in the rates of the cost items compared to market trends. After conducting positive due diligence and value for money assessment, the MoH signed a commercial contract with each of the 5 bidders on 5 February 2015.

**Figure 2:**
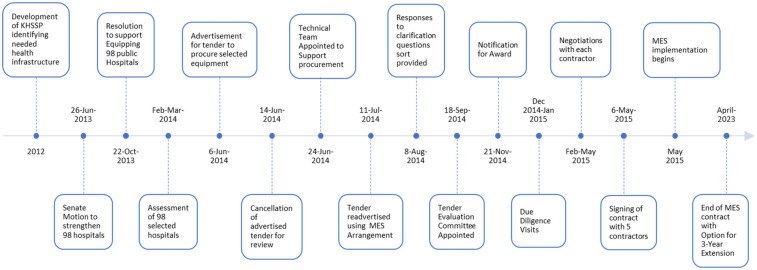
Schematic illustrating timelines of MES procurement and implementation process.

Between February and May 2015, a multi-disciplinary team of legal, medical, financial, and insurance experts conducted dialogue sessions with each of the 5 bidders. The purpose of the dialogue sessions was to ensure that the contract aligned with standard legal, financial, and technical requirements and clarify the expectations on all the technical schedules, particularly implementation and scope of service schedules. Insurance coverage requirements and performance measurements to monitor contract performance were also agreed upon. After successful dialogue sessions, a fully effective contract for implementation was signed on 6 May 2015 between the MoH and each successful bidder. Figure 2 shows the timelines of MES procurement and implementation processes.

### 2.5 MES implementation

The implementation of the MES contract had two phases. Phase 1 involved the supply, installation, commissioning, renovation, and fit-out work for buildings where equipment was to be installed. Before installing each piece of equipment, comprehensive interior and exterior renovations were undertaken by each contractor to upgrade the buildings, finishings, and fittings related to where each target service was to be set up. Personnel training was conducted per the training schedule defined for the cadre(s), numbers to be trained, training type, scope, and duration. Three types of training were provided: on-the-job user training for all the personnel interfacing with the equipment, maintenance training for biomedical engineers, and special training primarily for dialysis, ICU, and imaging personnel. As dialysis and ICU services were being established in the selected hospitals for the first time, a comprehensive 4-month training program was provided for nurses with general nursing training to prepare them for postings in dialysis units and ICU services. An intensive 2-week clinical training for digital imaging systems, mainly on Doppler ultrasound, was also included to provide skills in the clinical interpretation of digital radiological images. The equipment installation and commissioning, renovations, and training phase were planned to be completed within a 1-year timeframe. If there was a delay in achieving an agreed activity in the implementation plan in a hospital, a delayed event penalty surcharge was applied.

After the MES service commissioning in each hospital, phase two began. This phase involved planned and unplanned equipment maintenance, ensuring that all equipment was maintained on an agreed uptime of 95% to 98%. Equipment replacement and decommissioning were also scheduled per the approved lifecycle replacement plan. Continuous training was also planned to be provided throughout the 7-year contract duration. Each contractor was to invoice the government 45 days after the end of each quarter. Payment for each invoice was due within 30 days based on the agreed schedule of quarterly payments.

### 2.6 Contract management

A procedure was implemented to manage and track the MES contract to ensure it remained on schedule. Distinct levels of management were established, including a Managed Equipment Services Implementation Committee made up of members from the Ministry of Health (MoH) who oversaw day-to-day management. This committee was to monitor planned activities in accordance with the contract and hold monthly meetings with each contractor to review progress. Each hospital also had a Hospital Managed Equipment Service Implementation Committee to supervise and report on MES implementation activities.

A Liaison Committee was established, consisting of three members from MoH and three from the contractor, responsible for resolving disputes and making recommendations to each party. An Investment Committee was also set up, comprising three members from MoH and three from each contractor, with the responsibility of reviewing the investment in MES equipment and services and having a forum for collaborative strategic discussions, particularly on equipment and contract variations for the efficient performance of the MES contract.

The government and contractor agreed on continuous feedback and communication to achieve MES goals. Monthly and quarterly self-monitoring performance reports were included to track achieved performance levels according to the agreed performance indicators. The monthly reports were discussed between the contractor and the MoH during scheduled meetings. The quarterly performance reports were attached to the quarterly invoice, which showed the performance failure deductions applied during the previous quarter.

Failure to meet the performance requirement by a contractor attracted a financial penalty, leading to a reduction of the quarterly fee paid to the contractor. The government's delay in quarterly payments also attracted a delayed payment penalty. In addition, failure to meet the performance requirements could lead to warning notices or eventual contract termination.

Each contractor was expected to have an effective and easily accessible toll-free automated help-desk communication system to document all telephone communications between the contractor and the health facilities. The help desk enabled the government to get a list of hospital calls reporting equipment failure, track the equipment's return to functional status, and compare it with the quarterly performance monitoring report and deductions applied.

## 3 Results

### 3.1 Outcomes of MES implementation

A total of 30 bids were received across different equipment categories: 23.3% for theatre, 26.7% for Central Sterile Services Departments (CSSD), 16.7% for laboratory equipment (Category 1), 6.7% for laboratory equipment (Category 2), 6.7% for renal services, 3.3% for intensive care units (ICUs), and 16.7% for imaging services.

Following the preliminary evaluation, 66.7% (20/30) of the bids were deemed non-compliant with the initial examination criteria. The most common reason for disqualification was non-adherence to the Original Equipment Manufacturer (OEM) criteria, which accounted for 43.3% (13/30) of the rejected bids. Additionally, 10% (3/30) of bidders failed to tender for all items within the specified lot, while 13.3% (4/30) did not provide the required tender security of 2% of the tender sum. Consequently, only 33.3% (10 out of 30) of the bids moved on to the technical evaluation stage, with 6 bidders (20%) successfully securing the award for supplying and installing equipment for operating theatres, CSSD, dialysis units, ICUs, and diagnostic imaging services under MES arrangements. However, the bidder for Laboratory Category 1 declined the offer letter, and no qualifying bids were received for Category 2, resulting in an overall contract success rate of 71.4%.

As a result of these contracts, the five OEMs supplied and installed a total of 8,613 medical equipment across 98 hospitals enhancing access to specialised medical services. Table 1 summarises the procurement and deployment of MES equipment across healthcare facilities across the country.

**Table 1:** Summary of MES procurement and deployment.

Lot	Equipment category	Units (n)	Hospitals (n)	Deployment details
1	Operating theatre	1,479	98	191 theatres established
2	CSSD	3,637	96	96 units established
3	Laboratory (Cat. 1)	N/A	N/A	No eligible OEMs
4	Laboratory (Cat. 2)	N/A	N/A	No eligible OEMs
5	Dialysis	833	49	49 units established, each with 5 stations
6	ICU	2,079	11	11 ICUs established, each with 6 ICU & 3 HDU beds
7	x-ray (fixed)	585	98	100 units established
	x-ray (mobile)		98	98 units
	Ultrasound		96	96 units established
	Mammography		50	50 units established
	OPG x-ray		49	49 units established

The equipment was utilised to outfit 191 theatres in 98 hospitals, 96 CSSD units in 96 facilities, and 49 dialysis units, each with 5 dialysis stations, as well as 11 intensive care units, each comprising 6 ICU beds and 3 HDU beds across 11 hospitals. In diagnostic imaging, 98 diagnostic x-ray units were established, together with 96 ultrasound units, 50 mammography units, and 49 orthopantomography (OPG) x-ray units.

**Figure 3:**
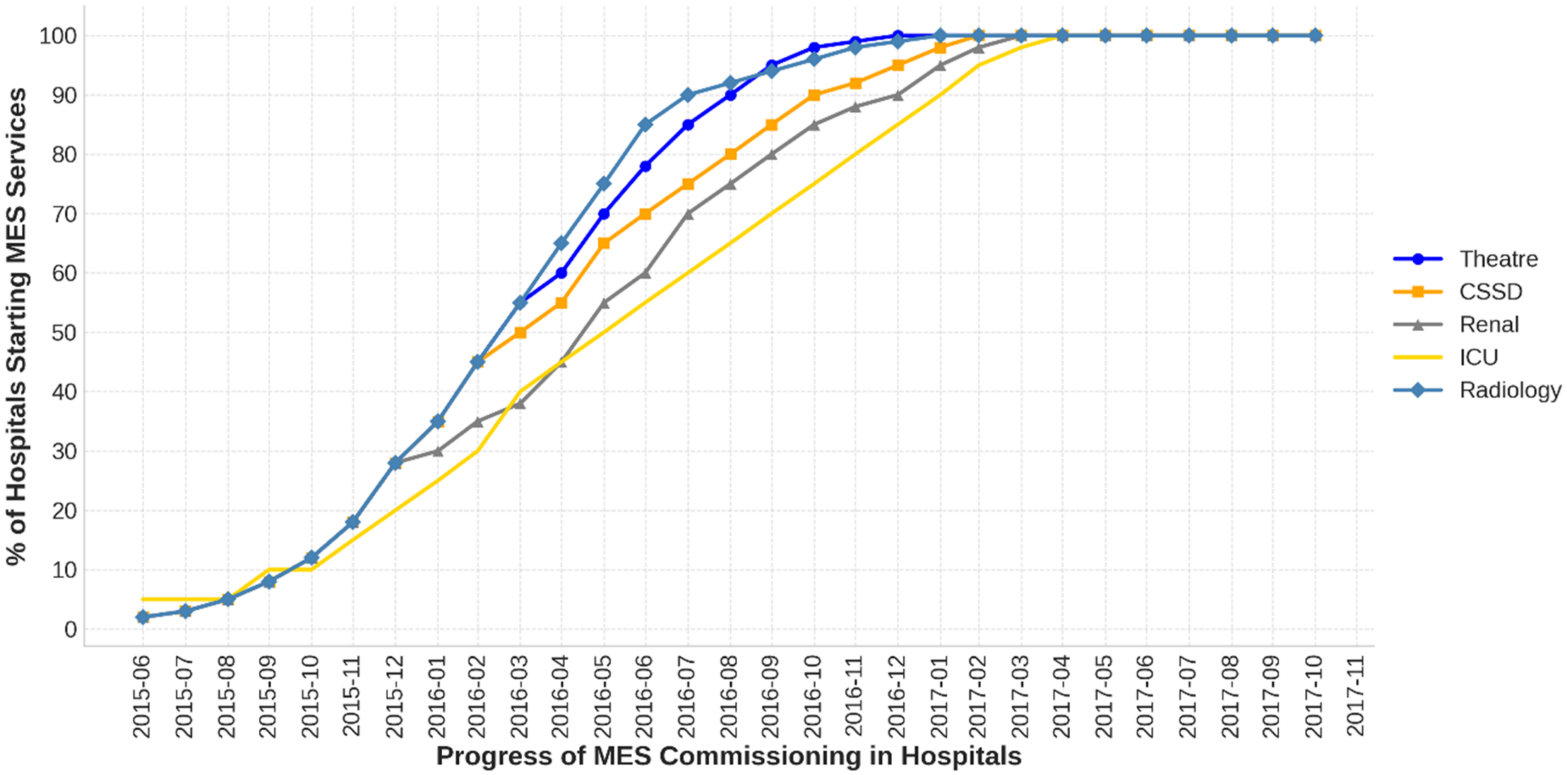
Timeline of MES service commissioning in hospitals.

Figure 3 illustrates the progress of MES implementation in 98 hospitals, highlighting delays across all contractors beyond the planned 12-month completion timeframe. Within 12 months of signing the MES contract in June 2015, the installation and service commencement rates were 68% for theatres, 76% for CSSD, 50% for dialysis, 27% for ICU, and 82% for radiology.

Completion timelines exceeded the projected 12-month period, with CSSD reaching 100% completion in August 2016, reflecting a delay of three months. Radiology services were fully implemented by December 2016, with a seven-month delay, while ICU completion was achieved in February 2017, nine months behind schedule. Dialysis services were completed in December 2017, 19 months late, while theatre equipment installation remained incomplete at 2 hospitals.

**Figure 4:**
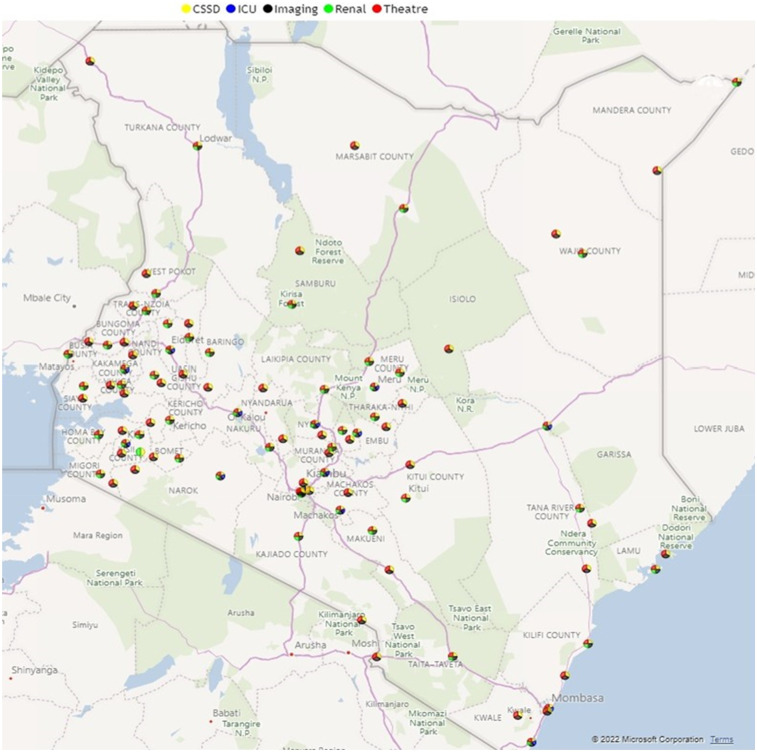
Geographical distribution of managed equipment services in Kenya.

Following the commissioning of MES services, the geographical distribution and accessibility of theatre, CSSD, dialysis, ICU, and diagnostic imaging services are illustrated in Figure 4, which shows the geographical distribution of these services in Kenya.

### 3.2 Lessons learned

The current MES arrangement is the first of its kind in Kenya. After completing the planning, procurement, and implementation processes, we have reflected on the lessons learned. We have presented these lessons based on their importance, irrespective of the MES planning, procurement, and implementation process sequence. We have also highlighted positive and negative elements and mitigation strategies where appropriate.

Putting the MES arrangement in place was a complex task for several reasons. Firstly, the number of hospitals selected for MES implementation was extensive, involving 98 hospitals and five different services that used countless pieces of equipment. Secondly, preparing equipment technical specifications and scope of services for each contractor, selecting the contractors, and engaging with each contractor to agree on performance standards required to deliver each healthcare service took considerable time and diverse skills. Thirdly, from the beginning to the MES contract signing, it took one year of intensive work, with three months of dialogue sessions with the contractors. Therefore, based on our experience, we recommend allocating sufficient time to assess the hospitals, prepare required documentation, and conduct dialogue sessions with the selected bidder(s).

The procurement process of MES faced a high bidder disqualification rate, likely due to a lack of clear understanding of MES arrangements from bidders. Additionally, no laboratory bidders were selected for laboratory categories 1 and 2 because the bidders did not form joint ventures that could meet all required equipment specifications for each laboratory component. This was a significant shortfall, as laboratory services are pivotal in patients management. In hindsight, structuring laboratory procurement into smaller, department-specific lots could have attracted more bidders, though this would have increased the complexity of evaluation and contract management. Future MES initiatives should consider a hybrid approach that balances lot size with bidder participation feasibility.

Each contractor had developed an implementation plan defining the tasks and a timeframe for the completion of each task for each hospital. The MES contract set a contractual timeframe of one year to commission all MES hospitals. Any task completed after the agreed timeframe was categorised as delayed. Despite this, a delay occurred due to the hospitals' buildings being old with architecture and the in-place electrical, ventilation, air conditioning, and plumbing systems needing to be fixed to accommodate the installation of modern equipment. Specifically, dialysis and ICU required significant remodelling and fit-out work to meet their standards and designs. Additionally, 13.3% of the hospitals needed new buildings to accommodate diagnostic imaging. The cause of the delay was related to the inadequate hospital assessment conducted before bid submission by the interested bidders to identify the infrastructure and utility requirements for each piece of equipment. The insufficient evaluation of the pre-bid also underestimated the renovation required, significantly impacting the contractor's expenditure. Due to the contractor's inadequate pre-bid hospital assessments, which were essential for determining the scope of necessary renovations and fit-out work, the government was unable to cover the additional renovation costs. Therefore, it is recommended to include an architect in the hospital's assessment prior to the MES design phase. The architect could guide the extent of required remodelling and fit-out work, estimate renovation costs, and advise on whether to renovate or construct a new building. The assessment results can be incorporated into the tender documents.

There were delays in several hospitals due to water availability and quality and electricity challenges, which affected the start-up of diverse services such as theatre, CSSD, dialysis, and imaging. Water unavailability and inadequate quality affected the use of theatres, sterilisation, and dialysis during the start-up phase. Additionally, hard water causes limescale build-up, which affects the performance of sterilisation and autoclaves, and the reverse osmosis membranes used in dialysis, leading to unnecessary expenses due to frequent replacements. Also, some hospitals' lack of 3-phase power caused delays in using fixed digital x-rays. Targeted interventions resolved most issues related to water unavailability and 3-phase power challenges in many hospitals. However, water quality remained a challenge in several hospitals, indicating the need for a water treatment plant. It is recommended that water treatment plants be included as a mandatory MES infrastructure requirement for dialysis and CSSD to ensure sustainability.

Rapid installation of new medical equipment in hospitals brought the challenge of recruiting and onboarding new personnel to use the equipment to deliver the newly established services. As a result, training and recruiting additional personnel with the required skills was needed. This required hospitals to seek additional funding for salaries and associated costs of new employment opportunities. For example, only 25 radiologists were available in 25.6% of MES-targeted hospitals. Although there were 260 radiographers in 47 counties, none were allocated to 20.4% of the MES hospitals, requiring their redistribution to cover all MES hospitals. The digitalisation of imaging equipment enabled the digital transmission of images from hospitals with no radiologists to hospitals without radiologists for reporting on x-ray images, even in rural areas where qualified radiologists were not readily available. In addition, there were few hospitals with ICU and dialysis services in our settings before MES implementation. Therefore, ICU and renal nurses' training had yet to be prioritised, leading to a limited pool of nurses available for deployment to the newly established ICU and renal centres. The MES contract had to structure a 4-month short-term specialised training for nurses with general nursing training, beginning with 4 nurses for each centre being trained on a rotation of 2 nurses for a 2-month cycle at a time to ramp up the number of nurses required to begin running new dialysis and ICU services. To address the gaps, it is recommended that future MES arrangements include workforce planning, structured personnel training, and funding mechanisms for human resource development.

Even after starting up MES-supported services, the availability of consumables remains critical to the continued delivery of services. Unfortunately, the MES contract excluded consumables, with the obligation of consumables remaining with respective hospitals. As a result, across the spectrum of MES-supported hospitals, interruptions of services continued to occur due to frequent stock-out of essential consumables, leading to patients being unable to access required services. Therefore, in hindsight, we recommend bundling consumables with other MES services and confining the hospital to provide only personnel and utilities.

The MES contract defined performance parameters and monitoring methods to measure and track the service(s) performance. Related service failure types linked to service failure points were used to calculate the financial deduction. Each contractor was expected to self-monitor, report on service failure type, and calculate service failure points and applicable financial deductions using a defined formula at the end of every quarter. MoH's role was to ensure that the MES contractor complied with all the performance parameters described, validating the accuracy of the performance reports received. This included ensuring that equipment was maintained and serviced per the agreed schedule and rectified where equipment-related faults occurred within the agreed period. Monitoring or tracking how well distinct MES aspects are performing is essential in ensuring that each party is adhering to its obligations. A financial cost is applied to the contractor for performance failures and service unavailability and to the procuring entity for the delay in quarterly payment. Although self-monitoring has worked well, it requires a high level of ability to self-regulate and transparency. Therefore, we recommend setting aside financial resources for outsourcing monitoring activities to ensure contractors perform their duties and appropriate deductions are applied.

A gain-share arrangement was incorporated into the contract to ensure that cost savings or efficiency improvements benefited both the contractor and the Ministry of Health (MoH). Under this arrangement, gains such as cost savings from improved labour efficiency, lower interest rates, or insurance refunds related to equipment losses were shared in the form of additional equipment or personnel training. However, the calculation of these gains relied on contractor-determined figures, raising concerns about transparency and independent verification. Gains were assessed and distributed at two points during the contract period. Future MES agreements should enhance the transparency and accountability of the gain-share mechanism by establishing standardised, independently verifiable calculation methods to maximise financial efficiency and ensure equitable benefit distribution.

Securing broad stakeholder support for MES in Kenya has been challenging. While health professionals have largely embraced the initiative, other key stakeholders have shown limited support. As a large-scale and high-impact undertaking, MES would have benefited from a structured approach to stakeholder identification, influence assessment, and targeted engagement to build consensus. The lack of a clear engagement strategy contributed to misunderstandings about the complexity and cost structure of MES, fuelling perceptions of inadequate value for money and leading to its politicisation. Given the scale and strategic importance of MES, the absence of a robust stakeholder engagement plan was a significant shortcoming. Future MES projects should incorporate comprehensive stakeholder mapping and proactive engagement strategies to ensure sustained support, address concerns early, and minimise political resistance.

## 4 Discussions

We have described in detail the planning, procurement, and implementation processes of a large-scale MES arrangement in Kenya, offering variable insights for similar initiatives. The findings demonstrate that a well-planned and well-implemented MES arrangement can enhance specialised healthcare services, even in an environment with fiscal constraints. However, MES implementation is inherently complex requiring strong governance, a highly skilled multidisciplinary team, and meticulous planning to navigate logistical, financial and operational challenges.

A critical factor in MES success is adequate time allocation for planning, and conducting a well-detailed hospital assessment before designing procurement, infrastructure design and contract negotiations. In our settings, gaps in facility readiness, staffing and equipment compatibility with the available space caused implementation delays, underscoring the need for thorough preparatory work. A structured implementation roadmap with clear timelines and hospital specific requirements can improve accountability and efficiency. Early stage risk assessments are essential in identifying potential bottlenecks such as utility and infrastructural constraints and personnel requirements that may cause delays in implementation. Kenya's experience highlights the importance of conducting comprehensive hospital assessments before designing an MES solution to ensure infrastructure, utilities, and human resource requirements align with service expansion goals.

Contract design and enforcement play a critical role in determining MES effectiveness. The Kenyan MES model incorporated a penalty clause to enforce performance standards, which encouraged compliance and reduced the need for intensive oversight. However, contract enforcement mechanisms require active monitoring to prevent inefficiencies. Week oversight has led to performance failures in other PPPs, where the private sector has underdelivered due to inadequate accountability and governance structures. Penalties have been known to be effective in discouraging opportunistic behaviour and can minimise the supervision required during the operational phase of MES (42, 43). To avoid similar pitfalls, MES arrangements must include robust governance frameworks, clear contractual obligations, and strong oversight institutions to ensure service delivery aligns with agreed terms.

Stakeholder engagement is another critical determinant of MES success. The implementation process requires the involvement of multiple government agencies, healthcare providers, private contractors, and surrounding communities. While broad participation enhances project acceptance and ensures smoother execution, it can also introduce complexities. Diverse interests may lead to differing views and conflicts, delays, or resistance to change. The Kenyan experience suggests that proactive engagement—where stakeholders' concerns are addressed through transparent communication—can enhance buy-in and minimise opposition. However, in some cases, stakeholder fragmentation has stalled PPPs, particularly when political and bureaucratic conflict with PPP objectives (44, 45). Effective MES implementation, therefore, requires deliberate strategies to align stakeholder priorities and mitigate resistance.

One of the significant challenges in implementing MES in Kenya was the shortage of specialised personnel (46, 47). The rapid expansion of MES-supported healthcare services outpaced available trained personnel, particularly in dialysis and ICU care. Structured short-term training for nurses with general nursing training provided a temporary solution, but long-term sustainability requires more robust workforce planning. Similar workforce shortages have been observed in other MES and PPP healthcare projects globally. Numerous studies have also demonstrated that structured short courses can enhance nurses' knowledge and skills in areas such as intensive care while also contributing to the country's ability to provide much-needed services (48). To prevent service disruptions, MES contracts should include provisions for continuous training, skills transfer, and workforce development. Additionally, leveraging technological solutions such as teleradiology, as implemented in Kenya and elsewhere, can address specialist shortages by enabling remote diagnostic services (49, 50).

The MES arrangements in Kenya rapidly expanded access to specialised healthcare services across all 47 counties, significantly reducing the need for patients to travel long distances for critical services. By shifting maintenance of equipment to the MES provider, minimising equipment downtime, and ensuring uninterrupted service delivery. Reducing service disruption has direct financial implications, lowering of out-of-pocket expenses for patients and reducing costly referrals to higher level facilities. Studies have shown that unchecked out-of-pocket payments can lead to increased poverty levels, delayed diagnosis, and poor health outcomes, particularly amongst the vulnerable populations (51, 52). Studies have also shown that the availability of working equipment improves healthcare workers' morale and patient outcomes (53). The Kenyan experience reinforces the importance of MES models in reducing healthcare inequities by decentralising specialised services.

International evidence supports the potential benefits of PPP in healthcare (54). A well-structured MES arrangement can also reduce long-term costs. Research from MES projects in high-income countries suggests that outsourcing medical equipment management can yield cost savings of 5%–10% compared to public-sector-managed procurement (55, 56). These global experiences demonstrate that MES can be a viable strategy for healthcare expansion, provided contracts are structured with clear service-level agreements and performance monitoring mechanisms.

Despite the valuable insights gained from this study, several limitations must be acknowledged. First, the needs assessment process relied primarily on administrative consultations rather than a systematic evaluation of healthcare demand. This approach may have led to resource misallocation, as facility needs were determined based on institutional perspectives rather than comprehensive epidemiological and demographic analyses. A more data-driven assessment could have provided a clearer understanding of service gaps and equipment distribution priorities.

Second, the procurement process was heavily influenced by legal and procedural compliance, which, while ensuring adherence to regulations, resulted in a high exclusion rate of bidders. This limited competition and may have increased costs or restricted access to potentially superior equipment and service providers. Future MES initiatives should balance regulatory requirements with procurement efficiency to ensure value for money.

Third, this study focused on Kenya's MES experience, which may limit the generalisability of the findings to other contexts. Differences in regulatory frameworks, healthcare financing structures, and political environments could affect how MES arrangements are implemented elsewhere. While international comparisons provide useful parallels, direct application of Kenya's model to other LMICs should be approached with caution, with necessary adaptations to fit local conditions.

Finally, the study is primarily focused on MES implementation, with limited insight into long-term sustainability. While early findings suggest improved healthcare access and reduced equipment downtime, the durability of these benefits remains uncertain. Issues such as contract renegotiations, maintenance challenges, and equipment obsolescence will require further research to assess MES effectiveness over extended periods. A more comprehensive evaluation involving field surveys, direct hospital assessments, and patient outcome data would provide a more robust analysis of MES impact on healthcare delivery.

## 5 Conclusion

The learning from implementing MES in Kenya highlights both its potential benefits and critical challenges. By integrating equipment procurement, installation, training, maintenance, and replacement, MES has improved service readiness while reducing upfront costs and ensuring long-term functionality. However, challenges such as implementation delays, infrastructural constraints, and training gaps underscore the need for strategic planning, robust governance, and transparent contracting. As LMICs consider MES as a model for healthcare expansion, Kenya's experience provides critical lessons. Strengthening needs assessments, procurement processes, and long-term impact evaluations will be essential in refining MES strategies and ensuring sustainable healthcare improvements.

## Data Availability

The raw data supporting the conclusions of this article will be made available by the authors, without undue reservation.
